# Revealing the Diversity and Quantity of Peritrich Ciliates in Environmental Samples Using Specific Primer-based PCR and Quantitative PCR

**DOI:** 10.1264/jsme2.ME12056

**Published:** 2012-10-26

**Authors:** Xihan Liu, Jun Gong

**Affiliations:** 1 Laboratory of Environmental Microbiology, Yantai Institute of Coastal Zone Research, Chinese Academy of Sciences, Yantai 264003, China; 2 Key Laboratory of Coastal Zone Environmental Processes Yantai Institute of Coastal Zone Research, Chinese Academy of Sciences, Yantai 264003, China; 3 Institute of Evolution and Marine Diversity, Ocean University of China, Qingdao 266003, China

**Keywords:** Ciliophora, Peritricha, qPCR, specific primers, SSU rRNA gene copy number

## Abstract

Peritrichs are a diverse, ecologically important ciliate group usually with a complex life cycle. To date, the community of the peritrichs has been investigated by using morphology-based methods such as living observation and silver staining. Here we show a molecular approach for characterizing the diversity and quantity of free-living peritrichs in environmental samples. We newly designed four peritrich-specific primers targeting 18S rRNA genes that allow clone library construction, screening and analysis. A quantitative real-time PCR (qPCR) assay was developed to quantify peritrichs in environmental samples by using rDNA copy number as an indicator. DNA extracted from four water samples of contrasting environmental gradients was analysed. The results showed that the peritrich community was differentiated among these samples, and that the diversity decreased with the increase of water salinity. The qPCR results are consistent with the library sequence analysis in terms of quantity variations from sample to sample. The development of peritrich-specific primers, for the first time, for conventional PCR and qPCR assays, provides useful molecular tools for revealing the diversity and quantity of peritrich ciliates in environmental samples. Also, our study illustrates the potential of these molecular tools to ecological studies of other ciliate groups in diverse environments.

Peritrich ciliates are a large and highly diverse protistan group that so far comprises approximately 1,000 species (approximately 1/7 of all known ciliates) (28, 36, 37). Systematically, two monophyletic peritrich subclasses, the free-living *Peritrichia* (formerly the order *Sessilida*) and the parasitic *Mobilia* (formerly the order *Mobilida*), have been recognized (28, 44). As the larger group, the *Peritrichia* comprises about 105 genera (28) and at least 800 described species (16). Most of the free-living peritrichs have a complex life cycle with cyst, free-swimming (*i.e.* telotroch), and sessile stages (4, 28, 42). Since only the sessile forms are generally morphologically identifiable, they have been recorded from a variety of environments, *e.g.* freshwater (14), brackish (5, 41), marine (24, 37), and soils (13); from plankton (3), and biofilm or periphyton of immersed substrates and animals (12, 20, 22, 42).

The peritrichs are responsible for water clarification by their bacterivory; hence, they are an ecologically important group in aquatic environments (9, 17, 29). The abundance of peritrichs is not often reported, and when planktonic, is difficult to interpret because they are either attached to algae in the plankton, or are in stages of telotroch, which are usually difficult to identify (see (28) and references therein). There are records of the temporal and spatial distribution of peritrich populations (41), while the community ecology of free-living peritrichs has been little studied.

During previous decades, diversity, phylogenetic and ecological studies of protists have increasingly relied upon small subunit ribosomal RNA (18S rRNA) gene sequences (6). Because there are numerous 18S rRNA gene sequences from diverse organisms in public databases, specific PCR primers or probes can be designed (and tested *in silico*) for the taxa of interest. Using specific PCR primers (33) and the quantitative real-time PCR (qPCR) assay, both the diversity and quantity (indicated by rDNA copy number) of the specific taxa in environmental samples can be assessed efficiently (19, 34, 40, 45); however, a specific molecular tool for the detection and quantification of peritrichs from environmental samples is not yet available.

In this study, we aimed to design and evaluate PCR primers to amplify partial 18S rDNA of free-living peritrichs (subclass *Peritrichia* sensu Zhan *et al.* 2009) (44) and to select a primer set for qPCR that specifically quantifies peritrich rDNA copies in environmental samples.

## Materials and Methods

### Sampling

Water samples were collected from the Guangdang River, a small river in Yantai, China (37°28′N, 121°28′E), on August 15, 2011. The 10-km-long river originates from a freshwater reservoir and flows into the Yellow Sea, with a great salinity gradient. Sampling was made at four sites, one freshwater (F), two brackish (B1 and B2), and one marine (M). Two liters of surface water were sampled at each site in the lower tidal period, filtered through 200-μm-pore mesh, and taken back to the laboratory within 1 h. Subsamples were maintained at room temperature and examined for live peritrich ciliates under a stereoscope. Recognized cells were picked up with a micropipette and photographed *in vivo* under a microscope (BX51; Olympus, Tokyo, Japan). To collect eukaryotic biomass for molecular analysis, subsamples were gently filtered through 10-μm-pore polycarbonate membranes (diameter 47 mm; Millipore, Bedford, MA, USA) until filtration performance became obviously lower. The water volumes filtered were recorded as 220, 540, 480 and 240 mL for sites F, B1, B2 and M, respectively. The membranes were placed immediately into 2-mL cryotubes and preserved at −80°C for DNA extraction. Salinity, pH, water temperature, chlorophyll *a*, and dissolved oxygen concentration were measured *in situ* with a multi-parameter probe (MS5; Hach, Loveland, CO, USA) (Table S1).

### DNA extraction, primer design and PCR

Environmental DNA was extracted using the UltraClean Soil DNA Isolation Kit (MoBio, Solana, CA, USA). The concentration of the extracted DNA was checked using a NanoDrop 2000C spectrophotometer (Thermo, Winmington, DE, USA).

**Fig. 1:**
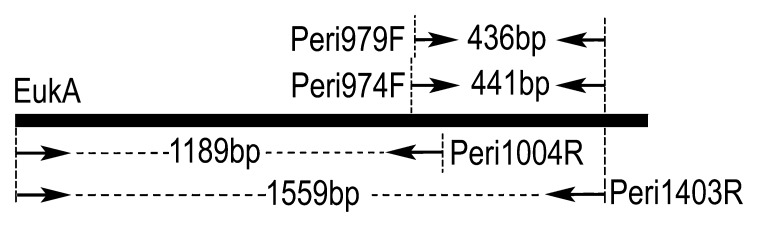
Schematic diagram of the rDNA region targeted by peritrich-specific primers designed in this study (*Opisthonecta henneguyi* (X56531) as the reference).

The design of specific primers for the subclass *Peritrichia* was based on alignment of the 18S rRNA gene sequences of 105 eukaryotic organisms (49 peritrichs, 36 non-peritrich ciliates, and 20 non-ciliate protozoa) retrieved from GenBank (NCBI). Four candidate peritrich-specific primers targeting the conserved regions were newly designed (Table 1). The two reverse primers (Peri1004R and Peri1403R) were paired with the eukaryote-specific primer EukA (30) to amplify longer fragments of 18S rDNA, and short fragments were amplified with two forward primers (Peri974F and Peri979F) (Fig. 1).

Primer specificity was checked by submitting the sequences to probeCheck (27) and evaluated against the GenBank database by *in silico* analyses using BLAST (1). Furthermore, the primers were experimentally evaluated by PCR using genomic DNA from peritrich isolates, with non-peritrich as control (Table S2). PCR was performed in a 25-μL reaction mixture, containing 0.2 mM dNTPs, 1.5 mM MgCl_2_, 0.4 mM of each primer, 1.25 units of *Taq* polymerase, and 1 μL DNA sample. The PCR program was carried out on a thermocycler (Biometra, Göttingen, Germany), consisting of 94°C for 4 min followed by 35 cycles at 94°C for 1 min, 50°C for 1 min and 72°C for 2 min, with an additional 7 min at 72°C. Amplicons were checked and separated by electrophoresis in 1% agarose gel containing Gelview (BioTeke, Beijing, China) in 0.5×TAE buffer and visualized under UV light. The size of bands was compared with a Trans2K DNA ladder (Transgen, Beijing, China).

### Cloning, RFLP analysis and sequencing

Since longer 18S rDNA fragments tend to reveal deeper classification, the primer set EukA/Peri1403R yielding a 1,600-bp fragment of 18S rRNA gene was chosen for clone library construction. Triplicate PCR products of each DNA sample extracted from the Guangdang River were combined to minimize PCR biases. The PCR products were gel-isolated and cleaned using the TIANprep Midi Purification Kit (Tiangen, Beijing, China) and cloned into competent cells of *Escherichia coli* with the InsTAclone PCR Cloning Kit (Fermentas, Hanover, MD, USA) according to the manufacturer’s instructions.

Initially, we used a clone library of the freshwater sample to test the specificity of the PCR using the primer set EukA/Peri1403R. Multiple white clones were randomly placed into 0.5-mL aliquots of Luria-Bertani broth, incubated overnight at 37°C, and sent to Sangon Company (Shanghai, China) for sequencing. The preliminary results showed that a considerable proportion of non-peritrich (*e.g.* algal) sequences were produced during PCR (Table 2).

In order to screen out these non-peritrich clones, we used a nested approach to perform colony PCR with the primer set Peri974F/Peri1403R against the randomly selected white clones from the libraries of all samples. Fifty white clones were randomly selected for colony PCR screening (with Peri974F/Peri1403R) and for sequencing (with M13F/R). Based on these data, we verified that the 11 clones positively amplified with Peri974F/Peri1403R did contain peritrich sequences, and that the 39 non-amplified did not. Of the four libraries, these verified clones were subsequently amplified with EukA/Peri1403R, and the PCR products were subjected to restriction fragment length polymorphism (RFLP) analysis with the restriction enzyme *Msp I* (Fermentas) according to the manufacturer’s instruction (Table 2). A few sequences subsequently determined to be chimeras were not counted. More than two clones of each RFLP type were randomly selected for sequencing on an ABI 377 automated sequencer (Sangon). Sequence chimeras were checked using Mallard (2), the Bellerophon server (23), and KeyDNATools (http://www.keydnatools.com/).

### Diversity and phylogenetic analyses

Peritrich sequences were aligned using Muscle 3.7 (10), and a distance matrix was generated with DNAdist using the Kimura 2-parameter model (11). Operational taxonomic units (OTUs) were determined with the software DOTUR 1.53 (35), with the furthest-neighbor algorithm and the 99% cutoff.

To examine the phylogenetic positions of the environmental peritrich sequences obtained, the closely related sequences identified by BLAST as well as the 18S rRNA genes of morphologically identified representatives were retrieved from GenBank. Sequences were aligned using Muscle, and a neighbor-joining (NJ) tree was constructed with MEGA 4.0 (38) based on the Kimura 2-parameter distance model. *Tetrahymena* species were taken as outgroup taxa.

### Quantitative real-time PCR assay

To quantify the peritrich rDNA copy numbers in the water samples, quantitative PCR was performed using SYBR Green with the primer set Peri974F/Peri1403R, which amplifies a fragment of about 441 bp (Fig. 1). Serial tenfold dilutions (10^−1^ to 10^−8^) of a linear DNA fragment (21) were used to generate a standard curve. Using the primer set M13F/M13R, this 1775-bp-long linear fragment was obtained from PCR amplification of a circular plasmid (pTZ57R/T vector; Fermentas), which contained an insert of the EukA/Peri1403R fragment of a *Vorticella* sp. (accession no. JQ743703). The 25 μL reactions contained 12.5 μL Maxima SYBR Green PCR/Rox qPCR Master Mix (Fermentas), 0.4 μM of each primer, 1 μL template DNA, 0.1 μLbovine serum albumin (BSA, 100 μg μL^−1^) and 10.4 μL RNase-free water. All reactions were performed in triplicate with an ABI 7500 Fast Real-Time PCR System (Applied Biosystems, Foster City, CA, USA). The PCR program followed the three-step protocol, starting with an initial soaking step at 50°C for 2 min and activation of Maxima Hot Start *Taq* DNA Polymerase at 95°C for 10 min, followed by 40 cycles of denaturation at 95°C for 15 s, annealing at 60°C for 30 s, and extension at 72°C for 30 s. Data collection time was set at the extension step. The specificity of the PCR product and the most fitted annealing temperature were verified according to melting curve analysis (preprogrammed as follows: 95°C for 15 s, 60°C for 1 min, 95°C for 30 s, and 60°C for 15 s). The efficiency of the amplification was calculated as follows: E = (10^−1/k^ −1) × 100%, where E is PCR efficiency and k is the slope. The number of molecules amplified in the standard was calculated using the following formula: molecules μL^−1^ = *a*/(1,775 × 660) × 6.022 × 10^23^, where *a* is the DNA concentration of the standard (g μL^−1^), 1,775 is the fragment length, 660 is the average molecular weight of one base pair of double-strand DNA, and 6.022 × 10^23^ is the molar constant (40, 45).

### Nucleotide sequence accession numbers

Environmental 18S rRNA gene sequences of peritrichs have been deposited in the GenBank database under accession numbers JQ743680–JQ743702.

## Results

### Specificity of newly designed primers

The *in silico* check using probeCheck and BLAST against GenBank confirmed that none of these newly designed primers matched members of the subclass *Mobilia* and other organisms, and that all the peritrich 18S rDNA sequences available at present were targeted very well across lineages except for a few species. Peri974F and/or Peri979F matched perfectly with 46 (93.9%) peritrich sequences belonging to genera such as *Astylozoon*, *Carchesium*, *Epicarchesium*, *Epistylis*, *Ophrydium*, *Opisthonecta*, *Pseudovorticella*, *Telotrochidium*, *Vaginicola*, *Vorticella*, *Zoothamnium* and *Zoothamnopsis*, but mismatched with three peritrich sequences at 2–4 sites: *Epistylis galea* (AF401527), *Campanella umbellaria* (AF401524), and *Opercularia microdiscum* (AF401525). Non-peritrich sequences differed from the primer at at least 3 sites. Peri1004R showed perfect matches with 7 peritrich sequences, 1 mismatch at the 5′ primer end with 39 peritrich sequences, and 3–4 mismatches with *E. galea* (AF401527), *C. umbellaria* (AF401524), and more than 4 mismatches with *O. microdiscum* (AF401525) and non-peritriches. Peri1403R showed perfect matches with 47 peritrich sequences including *E. galea* (AF401527), *C. umbellaria* (AF401524), but showed 1 mismatch with *O. microdiscum* at the 5′ primer end and an uncultured bacterium (DQ298151) at the 3′ primer end, and no less than 3 sites for other non-peritrich sequences.

The primer pairs EukA/Peri1004R, EukA/Peri1403R, Peri974F/Peri1403R and Peri979F/Peri1403R all produced positive amplifications on genomic DNAs of peritrich isolates, and generally non-amplification of non-peritrich ciliates (Table S2). One microliter of template DNA or about 1/20 of genomic DNA of a single isolated peritrich cell of *Vorticella* sp. was enough to yield bright specific bands with 35 PCR cycles (data not shown). Using cloning and sequencing we also confirmed that the primer set Peri974F/Peri1403R was highly peritrich-specific, hence it is suitable for qPCR and reliable for environmental surveys (see Materials and methods).

Compared with Peri974F/Peri1403R, PCR with the primer set EukA/Peri1403R produced a longer fragment of 18S rDNA, but its specificity was much lower, as less than 10% clones examined (*n*=697) were peritrichs (Table 2). Nevertheless, it was shown that peritrich-specific clones can be successfully screened out by primers Peri974F/Peri1403R whose amplicons are semi-nested within that of EukA/Peri1403R.

### Peritrich ciliate composition and distribution along the Guangdang River

**Fig. 2:**
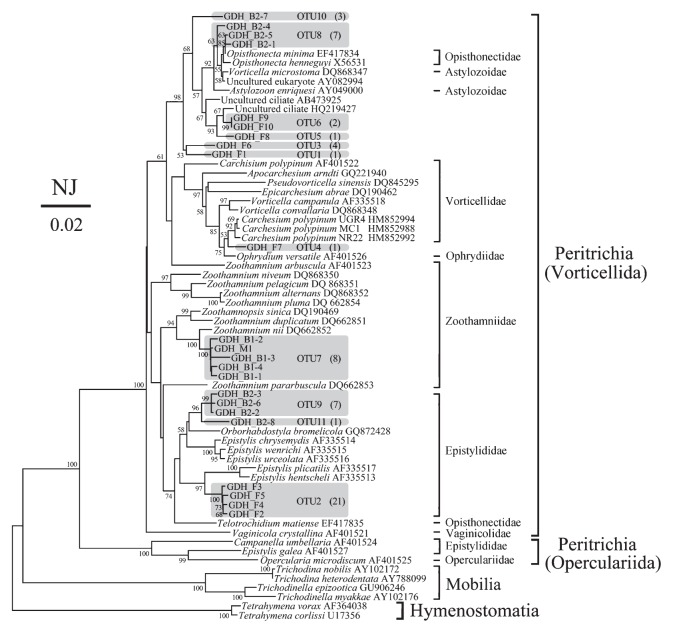
A Neighbor-Joining tree based on the Kimura 2-parameter distance model, showing the phylogenetic positions of the 11 peritrich OTUs from freshwater (F), two brackish (B1, B2), and marine (M) samples collected from the Guangdang River. Sequences obtained in this study are shaded. The numbers of clones belonging to each OTU are shown in the parentheses. Only bootstrap values no less than 50% are shown at nodes. *Vorticella microstoma*, which is placed apart from other congeners, is classified in the family Astylozoidae according to Sun *et al.* (2012). The scale bar indicates 2 substitutions per 100 nucleotide positions. Two *Tetrahymena* species are selected as outgroup taxa.

A total of 697 clones from 4 libraries of the river samples were screened, and 67 peritrich clones and 23 peritrich sequences were identified and obtained, 28% sequences were found to be chimeras, and 11 OTUs were finally determined (Table 2; Fig. 2). Peritrich OTUs were seldom shared among sites, except for one (OTU7) that was found at both sites B1 and M (Fig. 2). In the library of the freshwater site, 6 OTUs were detected; the most frequently found (70%) sequences belonged to the OTU2, which clustered with *Epistylis hentscheli* and *E. plicatilis* in the NJ tree; OTU3 was the second most abundant, accounting for 13% sequence abundance. OTU6 was clustered with an uncultured ciliate species (HQ219427) previously detected in a dimictic and eutrophic freshwater lake in France (31). Other OTUs (OTU1, 4 and 5) in the freshwater library were detected only once. Among these, OTU1 clustered with OTU3 at 53% support. OTU4 grouped with *Vorticellidae* (*Carchesium polypinium*) and *Ophrydiidae* with moderate bootstrap support (75%). The phylogenetic position of OTU5 was very close to OTU6.

For the two brackish sites, there were more OTUs (4 vs 1) in B2 than B1 (Table 2). OTU7 was represented by 7 clones in B1, clustering with *Zoothamnium nii* with 100% bootstrap support. The B2 library was dominated by OTU8 and OTU9 (relative abundance 39% and 39%), which likely belong to a population of *Opisthonecta minima* (EF417834, sequence similarity about 99%) and the family *Epistylididae*, respectively. OTU11 was similar to OTU9 and belonged to *Epistylididae*. OTU10 was close to OTU1 and 3. Among these OTUs, the taxonomic positions of OTU1, 3 and 10 could not be resolved in our phylogenetic analyses.

For the morphological observation of water samples, we only found two morphospecies *Epistylis* sp. and *Zoothamnium* sp. at freshwater and brackish sites, respectively (Fig. S1).

### Quantifying peritrich ciliates using rDNA copy numbers as an indicator

**Fig. 3:**
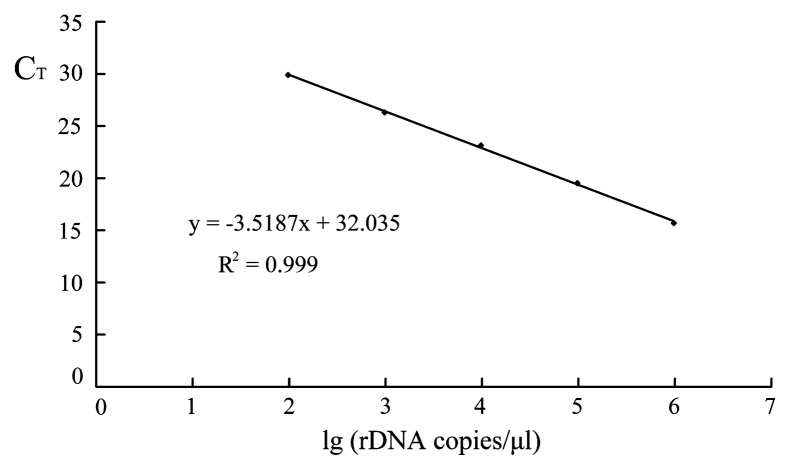
Standard curve showing the linear relationship between C_T_ values and lg rDNA copy numbers for serially diluted 18S rDNA obtained from *Vorticella* sp. (accession no. JQ743703).

**Fig. 4:**
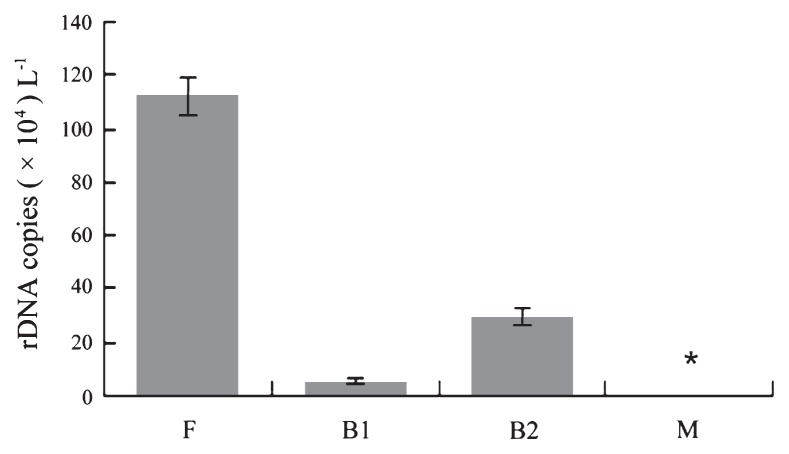
Peritrich rDNA copy numbers of four water samples. Error bars indicate the standard deviations. F, freshwater site; B1, B2, brackish sites 1 and 2; M, marine site. The asterisk indicates that no data could be obtained for the marine sample.

Specific PCR products were identified by melting curve analysis and a reproducible distinct melting point (Tm) of 78.39°C was observed from all standards. The linear relationship between the C_T_ (cycle threshold) and the log of rDNA copy number was C_T_=3.5187×lg (rDNA copies μL^−1^) + 32.035, with an amplification efficiency of 92.4% and R^2^ of 0.999 (Fig. 3). Melting curves of environmental samples were almost identical to those of the standards during the qPCR assay (Tm=79.97°C), except for the marine sample, for which melting curves with a peak of primer dimers were observed (Fig. S2). According to the C_T_ values obtained (23, 26, and 24), the peritrich rDNA gene copy numbers were calculated as 112.40±7.00, 5.72±1.08 and 29.91±3.30 (×10^4^ liter^−1^) in the F, B1 and B2 samples (Fig. 4). The non-specific amplicons of the marine samples were confirmed to be primer dimers by running electrophoresis in a 1.5% agarose gel (data not shown), so we could not quantify peritrich sequences in the marine sample.

According to the standard curve, the minimum detection sensitivity was 96 copies of 18S rDNA (C_T_ value of 29.97 ± 0.20), corresponding to rDNA extracted from 0.0006 cells of *Vorticella* sp., as we had estimated the rDNA copy number per cell of this species to be 160,000 (unpublished data).

Correlation among richness, abundance and environmental factors showed that water salinity was better coupled with peritrich OTU, sequence and rDNA copy numbers than other factors (Table S1). Their coefficients ranged from −0.81 to −0.90, indicating a negative effect of salinity on peritrich community diversity and abundance.

## Discussion

Although most ciliates including peritrichs can be easily observed under a microscope, their identification has been based on their morphology, infraciliature and silverline system revealed using silver staining methods (15), which, however, is difficult for non-specialists and time-consuming for ecological and biogeographic studies. Recent studies have also shown that morphological markers underestimate peritrich diversity (18, 43). This demonstrated the importance of using molecular tools to characterize the diversity of both isolated and environmental peritrichs.

Phylum-specific primers for ciliates have been developed and successfully used for soil ecosystems (8, 26, 33); however, for aquatic samples where highly diverse protists occur, some non-ciliates would also be targeted by these primers, resulting in substantial decreases of PCR specificity (8). In order to reveal both the diversity and quantity of ecologically important ciliate groups in aquatic environments, specific primers for ciliate taxa in lower ranks (*e.g.* classes, orders, families *etc.*) with highly specific performance may be one of the options. For example, order-specific primers have been used for studying clone library analysis (7) and denaturing gradient gel electrophoresis (DGGE) profiling (39) of marine planktonic oligotrich and choreotrich ciliates. In this study we extend and expand this strategy by designing and testing primers specifically and developed a qPCR assay for characterizing peritrich ciliates.

With the four samples from the Guangdang River, we showcase the good specificity of the primer pair Peri974F/Peri1403R, and the nested approach for studying peritrich community from environmental samples. It should be noted that species in the order *Operculariida* (*e.g. Epistylis galea*, *Campanella umbellaria*, and *Opercularia microdiscum*, see [43]; Fig. 2) have several mismatches mostly at the 5′ end with these primers. We did not detect any sequences closely related to or placed in this order, which is possibly a sign of the incapability of the primers to cover this group, or because no members of this group actually presented in the water samples. We presume that the latter is more likely since a relatively low annealing temperature (50°C) was employed in PCR; however, the primers need to be tested on isolated examples of the three species.

The pair EukA/Peri1403R amplifying a long fragment (about 1,600 bp) of 18S rDNA showed low specificity (<10%), as shown by the results of clone sequencing (Table 2). However, taking these clones with known sequence information as templates, we demonstrated that colony PCR with the primer set Peri974F/Peri1403R was only able to amplify the clones of peritrich sequences, not the non-peritrichs (*e.g.* alga). This suggests that the primer set Peri974F/Peri1403R outperforms EukA/Peri1403R in specifically recovering peritrich sequences from environmental DNA, although the length of PCR products is relatively shorter (about 441 bp).

The aims of this study were primer design and protocol optimization rather than application; however, the results provide an interesting insight into the diversity and composition of peritrich ciliates in water samples with distinct environmental gradients. With these peritrich-specific primers and sequencing, we detected 11 peritrich OTUs in about 1.5 L of water samples from the Guangdang River. The local (alpha-) diversity of peritrich ciliates seems to be low. This may be due to strong environmental gradients, especially salinity differences. Our data from the Guangdang River samples also showed that peritrich diversity generally decreased from the freshwater site to the marine site (Table 2, S1). Is peritrich diversity in water samples mainly controled by salinity, other environmental factors or food supply? Is peritrich diversity higher in freshwater than in marine habitats? These ecological/biogeographic questions warrant further studies with delicated experimental design and analyses.

From the Guangdang River samples we obtained several peritrich rRNA gene sequences (*e.g.* OTU1, 3, and 10), which are placed basal to known species/taxa with low bootstrap values (<50%) in the NJ tree. This does not necessarily mean that these sequences represent new species or new taxa of peritrichs, because among the 800 described peritrich species there is only a small proportion whose 18S rRNA genes are available from public databases. Nevertheless, it can be deduced that these OTUs could be rare in environmental samples and hence have frequently “escaped” morphological observation.

Quantitative PCR has advantages such as high accuracy and throughput and has been employed for quantifying eukaryotic groups such as diatoms, dinoflagellates, and picoeukaryotes (19, 34, 45). Using the primers Peri974F/Peri1403R, we have also developed a qPCR assay for quantifying peritrich rDNA copy numbers in environmental samples. The ratio of copy numbers generated using qPCR (116:5:32) is roughly consistent with the ratio of peritrich clone numbers (30:7:18) at least for the samples (F, B1 and B2) from the Guangdang River, although the water volumes filtered differed among samples (220, 540 and 480 mL). Furthermore, only 1 peritrich clone was detected from the 168 selected clones of library M, and the low abundance of peritrichs at this site was verified by the qPCR result (*i.e.* below the detection limit of qPCR). For the *Vorticella* sp. used for generating standard curves in the qPCR assay, we had estimated the rDNA copy number per cell to be 160,000 (more data on rDNA copy numbers from a variety of ciliate species are in preparation). The high sensitivity (lower detection limitation of about 0.0006 cells), specificity, reproducibility, and relatively low cost of using SYBR Green fluorescence enabled it become a practical tool for the detection and quantification of peritrichs in various environments.

The protocol for DNA extraction may be a key factor that affects the result of qPCR in our study. Some environmental DNA will inevitably be lost during the procedure in the use of either the UltraClean Kit or any other commercial kits for DNA extraction. This will lead to underestimation of the rDNA copy numbers, and hence the quantity of the taxa in question; however, the effect will be minimized if DNA samples are extracted and processed in parallel, and will be no problem in ecological studies in which many samples are compared.

With these peritrich-specific primers and the developed qPCR protocol, additional applications are possible. For instance, the internal transcribed spacers (ITS) and 28S rRNA genes have been suggested as barcodes for fungi, and for biogeographic analysis (32, 46). Our peritrich-specific forward primers can be paired with other primers targeting the ITS, 5.8S and 28S rRNA genes, thus yielding longer fragments which could be useful in population genetics or biogeographic studies. qPCR has also been used for assessing the gene copy number within a given genome (25, 40). Likewise, the qPCR assay could also be employed to assess rDNA copy numbers per peritrich cell from species to species, which might be helpful in calculating peritrich abundance in environmental samples given that the total rDNA copy number is known and the species composition of the sample is well identified.

In conclusion, we report here for the first time the development of specific primers for characterizing the peritrich ciliate community in environments using conventional PCR and real-time quantitative PCR assays. The DNA-based approach circumvents the difficulties of morphological identification and allows the detection of peritrichs of all development stages. The molecular approach also enables the analysis of many samples from different environments, which will undoubtedly stimulate further ecological and biogeographic researches on these organisms. Also, our study illustrates the potential of taxon-based molecular tools to reveal the diversity and quantity of other ciliate groups in freshwater, marine, and terrestrial ecosystems.

## Supplementary Material



## Figures and Tables

**Table 1:** Primers newly designed and tested for the amplification of peritrich 18S rRNA genes

Primer Name	Sequence (5′-3′)	Length	GC%	Tm (ºC)	References
EukA	AACCTGGTTGATCCTGCCAGT	21	52.4	58.9	Medlin *et al.*, 1988
Peri974F	GGAAACTCATCAGGRCAAGAAGATT	25	42	54.9–57.3	This study
Peri979F	CTCATCAGGRCAAGAAGATT	20	42.5	49.3–52.3	This study
Peri1004R	TCCTAYAATCTTCTTGYCCTGATG	24	41.7	51.7–56.6	This study
Peri1403R	GGGCGRTGTGTACATTTTG	19	50	51.8–54.5	This study

**Table 2:** Summary of the clone library analysis of the four sampling sites

Sampling site^b^	F	B1	B2	M
No. of clones checked	193	168	168	168
No. of RFLP types	19	11	11	1
No. of peritrich clones obtained	30	7	18	1
No. of peritrich sequences	10	4	8	1
No. of peritrich OTUs^a^	6	1	4	1

a OTU sequence similarity cutoff 99%.

b F, freshwater site; B1, B2, brackish sites 1 and 2; M, marine site.
