# Supplementary material for: Revealing the Diversity and Quantity of Peritrich Ciliates in Environmental Samples Using Specific Primer-based PCR and Quantitative PCR
Source: Microbes Environ. 2012 Oct 26;27(4):497–503. doi: 10.1264/jsme2.ME12056 (PMC4103560; doi:10.1264/jsme2.ME12056)
Supplement: Supplementary file 1 [file 27_497_s1.pdf]

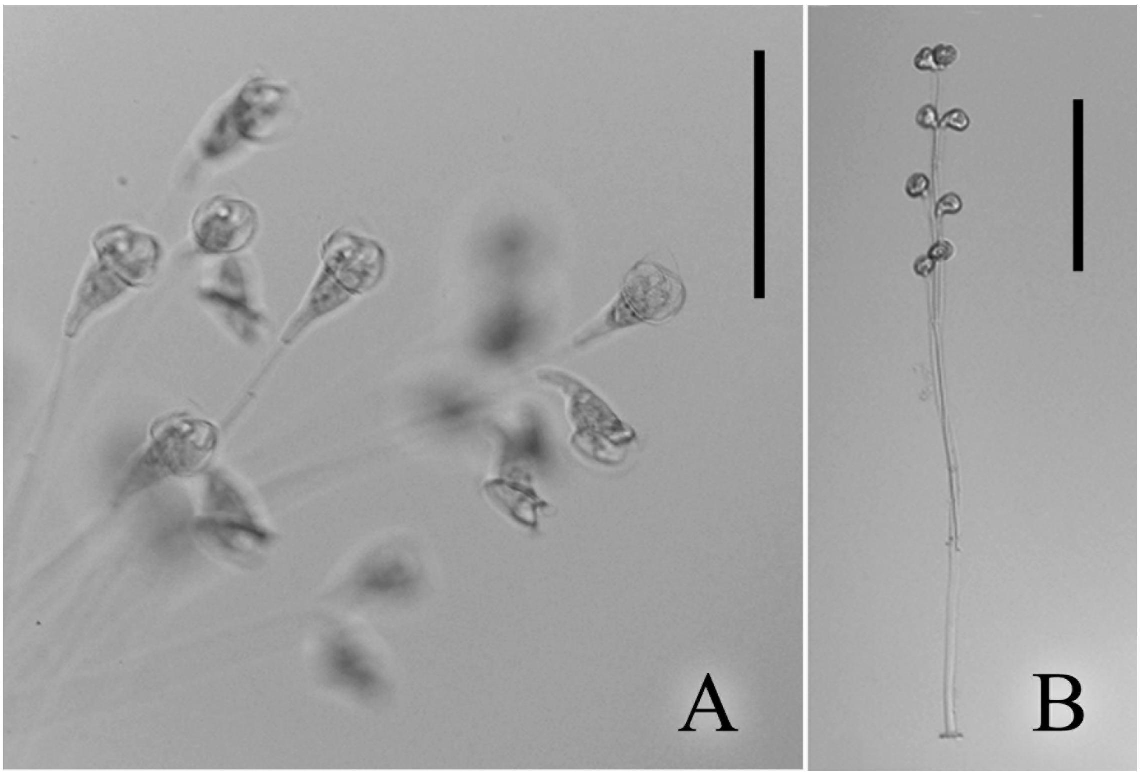

Fig. S1. Photomicrographs of peritrichs observed in water samples from the Guangdang river. A: *Epistylis* sp. B: *Zoothamnium* sp. Scale bars=50 µm.

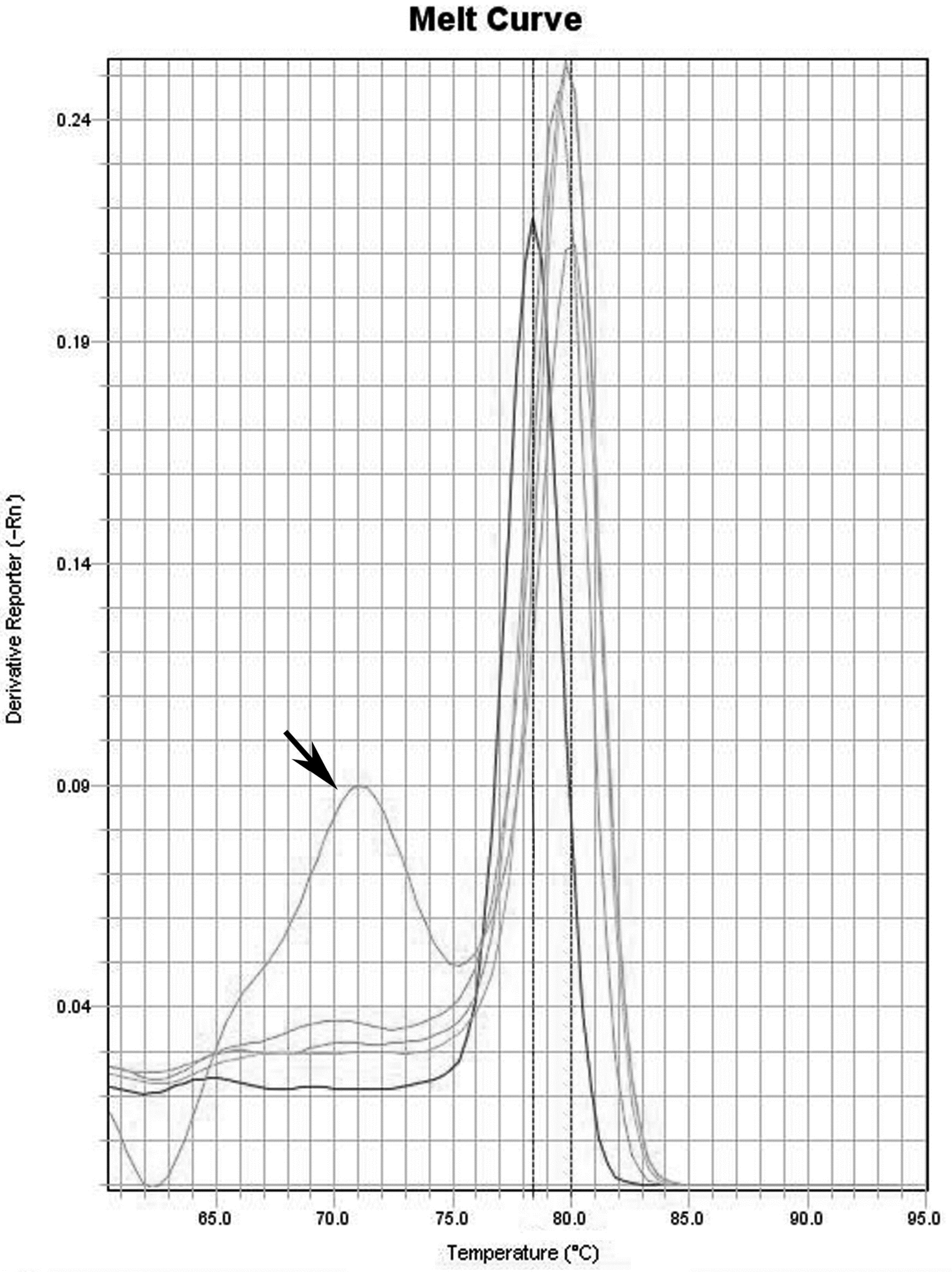

Fig. S2. Melting curve analysis of qPCR products. The arrow refers to the peak of the melt curve for the marine water sample, suggesting putative primer dimmers formed during the amplification. The melting temperatures of the linear standards and environmental samples were 78.39 °C and 79.97 °C, respectively.

Table S1. Environmental variables and their correlations with OTU, sequence and rDNA copy numbers.

	F	B1	B2	M	OTU number	Sequence number	rDNA copy number
Salinity (psu)	0.3	12.8	13.5	27.3	**-0.81**	**-0.90**	**-0.86**
pH	5.61	6.08	6.24	5.86	**-0.40**	**-0.18**	**-0.67**
Temperature (°C)	27.7	26.5	26.0	25.4	**0.74**	**0.78**	**-0.89**
Chlorophyll-*a* (μg/l)	9.44	6.63	5.96	11.24	**-0.08**	**-0.39**	**0.12**
Dissolved oxygen (mg/l)	6.54	7.66	5.83	7.34	**-0.75**	**-0.74**	**-0.47**

F, freshwater site; B1, B2, the brackish sites 1 and 2; M, the marine site. Pearson coefficients are highlighted in bold.

Table S2. Specificity of primer sets evaluated by PCR using genomic DNAs from peritrich and non-peritrich ciliates.

	EukA/Peri1403R	EukA/Peri1004R	Peri974F/Peri1403R	Peri979F/Peri1403R
Peritrichs				
Vorticella sp.1	+	+	+	+
Vorticella sp.2	+	+	+	+
Epistylis sp.	+	+	+	+
Zoothamnium sp.	+	+	+	+
Non-peritrichs				
Amphorellopsis sp.	-	-	-	-
Strombidinopsis sp.	-	-	-	-
Tintinnopsis sp.	-	-	-	-
Favella sp.	-	-	-	-
Strombidium sp.	-	-	-	-
Euplotes vannus	-	-	-	-
Pseudokeronopsis carnea	+/-	-	-	-
Paramecium caudatum	-	-	-	-

"+" means that a band was clearly presented, "-" means no band was observed, and "+/- " means a weak band was visible.
